# Institutional case volumes of thyroidectomies in Brazil and the impact of the COVID-19 pandemic: insights from a national database

**DOI:** 10.20945/2359-4292-2024-0152

**Published:** 2024-11-06

**Authors:** Leonardo Barbi Walter, Wallace Klein Schwengber, Anita Lavarda Scheinpflug, Andre Borsatto Zanella, Rafael Selbach Scheffel, Ana Luiza Maia, Jose Miguel Dora

**Affiliations:** 1 Universidade Federal do Rio Grande do Sul Faculdade de Medicina Hospital de Clínicas de Porto Alegre Porto Alegre RS Brasil Unidade de Tireoide, Serviço de Endocrinologia, Hospital de Clínicas de Porto Alegre, Faculdade de Medicina, Universidade Federal do Rio Grande do Sul, Porto Alegre, RS, Brasil; 2 Pontifícia Universidade Católica do Rio Grande do Sul Faculdade de Medicina Departamento de Medicina Porto Alegre RS Brasil Departamento de Medicina, Faculdade de Medicina, Pontifícia Universidade Católica do Rio Grande do Sul, Porto Alegre, RS, Brasil; 3 Universidade Federal do Rio Grande do Sul Instituto de Ciências Básicas da Saúde Departamento de Farmacologia Porto Alegre RS Brasil Departamento de Farmacologia, Instituto de Ciências Básicas da Saúde, Universidade Federal do Rio Grande do Sul, Porto Alegre, RS, Brasil

## Abstract

**Introduction:**

Providing widespread access to thyroidectomies while consolidating services in high-volume centers is a significant challenge in healthcare. In this context, from a national perspective, we aimed to analyze the impact of the COVID-19 pandemic on the institutional case volumes of thyroid surgery in Brazil.

**Material and methods:**

We analyzed retrospective thyroidectomy data from the Department of Informatics of the Unified Health System (Datasus), stratifying institutions into low-volume, intermediate-volume, and high-volume centers (<10, 10-100, and >100 thyroidectomies/year, respectively). We assessed the differences in absolute numbers and percentages of thyroidectomies performed during the pandemic years (2020-2022) compared with the pre-pandemic year (2019). Differences in the proportion of institutions based on case volumes from 2019 to 2022 were assessed using Cochran's Q test.

**Results and discussion:**

In 2019, 556 Brazilian institutions performed 15,331 thyroidectomies. Of these, 46.4% were categorized as low-volume, 48.4% as intermediate-volume, and 5.2% as high-volume institutions, accounting for 5.5%, 61.4%, and 33.1% of the thyroidectomies, respectively. Compared with 2019, the volume of thyroidectomies was lower by 41.2% in 2020, 37.0% in 2021, and 12.8% in 2022. When analyzing the proportions of institutions that maintained their pre-pandemic case volume in the first pandemic year, the intermediate and high-volume institutions experienced reductions of 34.9% (p < 0.001) and 58.6% (p < 0.001), respectively, while low-volume institutions presented a 4.3% reduction (p = 0.081).

**Conclusion:**

The COVID-19 pandemic disrupted the landscape of thyroidectomies in Brazil, particularly affecting intermediate-volume and high-volume institutions, while low-volume institutions showed greater resilience.

## INTRODUCTION

The challenge of providing widespread access to thyroidectomies across a broad area while consolidating services in high-volume centers is a significant issue in healthcare. Several studies demonstrate that surgical outcomes are influenced by case volume. Indeed, thyroid surgeries performed at high-volume institutions, compared with low-volume centers, are associated with decreased morbidity, mortality, and costs (1-3).

The management of patients with thyroid disease has been significantly impacted by the COVID-19 pandemic, leading to a notable decrease in diagnostic and treatment procedures in Brazil (4,5). In this context, it is uncertain how Brazilian institutions performing thyroidectomies responded to the COVID-19 pandemic from the perspective of the volume of cases handled at each center.

The Brazilian Unified Health System (*Sistema Único de Saúde* – SUS) operates on equity and universality of care principles. The SUS comprises three main components: public, private, and private health insurance subsectors. Most Brazilian health services are provided by the public subsector, which the government finances at the federal, state, and municipal levels for nearly 210 million inhabitants (6).

Data evaluating the institutional volume of thyroid surgeries for an entire country are scarce. Considering the vast dimensions of Brazil, achieving a balance between health access and quality of care is inherently complex, even during non-crisis periods. Therefore, this study aimed to evaluate the impact of the COVID-19 pandemic (2020-2022), compared with the pre-pandemic year (2019), on the institutional case volumes of thyroidectomies in Brazil.

## MATERIAL AND METHODS

### Study design and data source

We performed a retrospective study using 2019-2022 data from the Department of Informatics of the SUS (Datasus) Outpatient Information System. The collected data included the number and subtypes of thyroidectomies performed by institution, recorded using the following thyroidectomy Datasus codes: total thyroidectomy, 0402010043; total thyroidectomy with lymphadenectomy, 0402010051; and total thyroidectomy in oncology, 0416030270 and 041603270 (7). We defined 2019 as the pre-pandemic control year due to the impact of the COVID-19 pandemic on the diagnosis and treatment of thyroid cancer in Brazil after the beginning of 2020 (4).

### Outcome measures

For assessing case volumes, we adopted the cutoff values mostly used in previous studies (3), categorizing the institutions into three groups based on annual thyroidectomy volumes: low (<10/year), intermediate (10-100/year), and high (>100/year). We then analyzed the proportions of institutions and thyroidectomies within each volume category from 2019 to 2022.

### Statistical analysis

We assessed differences in the absolute number and percentages of thyroidectomies performed nationally and after classifying the institutions by case volume during the pandemic (2020-2022) compared with the pre-pandemic year (2019). Cochran's Q test was applied to analyze differences in the proportions of institutions classified by case volume, adjusted by the Bonferroni correction for multiple tests. For this final analysis, the institutions were dichotomized into those that maintained *versus* did not maintain the pre-pandemic classification during the COVID-19 pandemic years. P values < 0.05 were considered statistically significant, and all tests were two-tailed. Statistical analyses were performed using Statistical Package for Social Sciences (SPSS) software, version 25.0 (IBM Corp., Armonk, NY).

### Ethical considerations

The study project was approved by the institutional ethics committee (CAAE 29670919.9.0000.5327/GPPG 2019-0764). All authors vouched for the accuracy of the data and analyses.

## RESULTS

### Institutional case volumes of thyroidectomies in 2019 (pre-pandemic year)

A total of 556 institutions performed thyroidectomies in 2019 in Brazil. Of these, 258 (46.4%) were classified as low-volume, 269 (48.4%) as intermediate-volume, and 29 (5.2%) as high-volume institutions. That same year, 15,331 thyroid surgeries were conducted in the country, including 848 (5.5%) in low-volume, 9,404 (61.4%) in intermediate-volume, and 5,079 (33.1%) in high-volume institutions.

### Volume of thyroidectomies performed nationwide during the COVID-19 pandemic

The COVID-19 pandemic impacted the Brazilian healthcare system after April 2020, negatively affecting the number of thyroidectomies performed. Only 9,007 surgeries were performed in 2020, reflecting a 41.2% decrease compared with 2019. This decline persisted until 2021, with only 9,651 thyroidectomies performed, a 37.0% reduction from the pre-pandemic year. Although there was a slight improvement in 2022, with 13,369 surgeries performed, this surgical volume was still 12.8% lower than the one recorded in 2019.

### Volume of thyroidectomies performed by institutions during the COVID-19 pandemic

**Figure 1:**
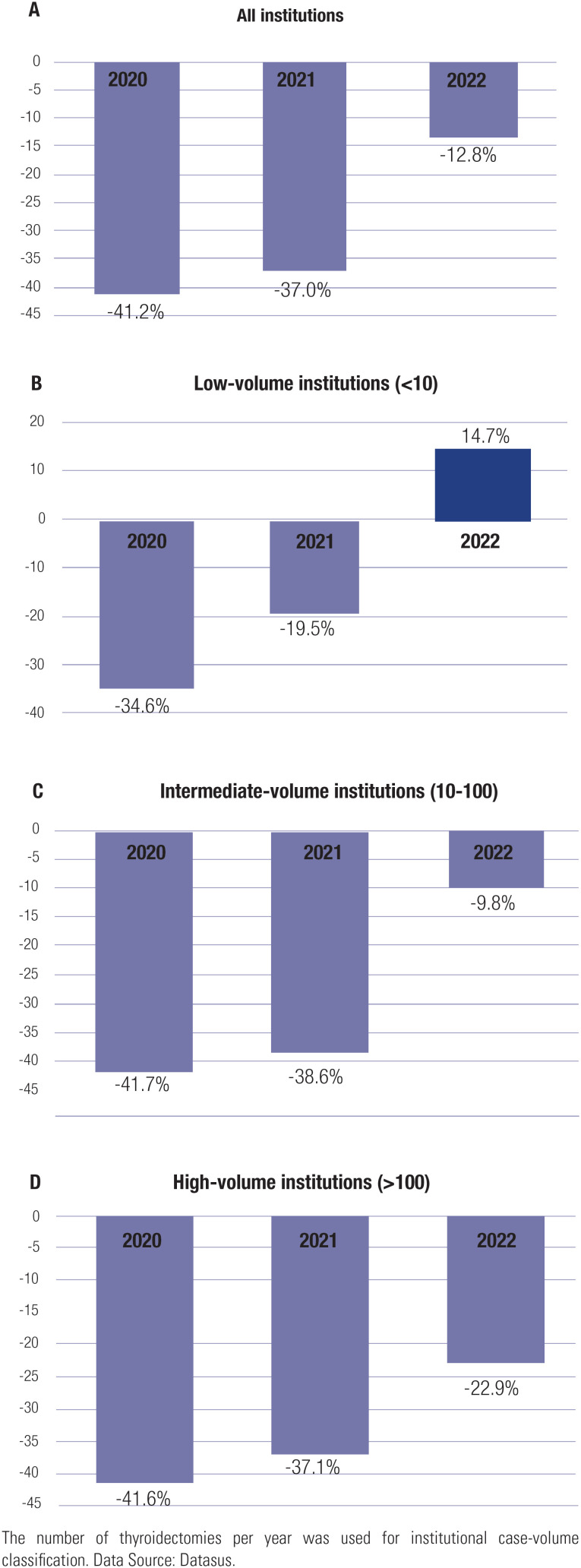
Differences in the numbers of thyroidectomies from 2020 to 2022 in Brazil, compared with pre-pandemic in 2019, for (**A**) all institutions, (**B**) low-volume institutions, (**C**) intermediate-volume institutions, and (**D**) high-volume institutions.

The number of thyroidectomies performed by low-volume institutions decreased from 848 in 2019 to 555 in 2020, a 34.6% reduction. In 2021, the number rebounded slightly to 683 but was still 19.5% lower than in 2019, and further increased to 973 in 2022, representing a 14.7% increase relative to 2019. In contrast, intermediate-volume and high-volume institutions did not return to their surgical volume during the pandemic years compared with 2019. Intermediate-volume institutions performed 9,404 thyroidectomies in 2019, 5,485 in 2020, 5,773 in 2021, and 8,482 in 2022, reflecting reductions of 41.7%, 38.6%, and 9.8%, respectively, compared with 2019. High-volume institutions performed 5,079 thyroidectomies in 2019, 2,967 in 2020, 3,195 in 2021, and 3,914 in 2022, reflecting reductions of 41.6%, 37.1%, and 22.9%, respectively, relative to 2019 (Figure 1).

The number of thyroidectomies per year was used for institutional case-volume classification. Data Source: Datasus.

### Proportions of institutions classified by case volume

To evaluate any significant differences in the proportions of institutions maintaining their case volume classification from the pre-pandemic year to the subsequent years (2020-2022), we conducted Cochran's Q test, applying the Bonferroni correction to adjust for multiple tests (Table 1).

**Table 1:** Cochran's Q test analyzing the proportion of institutions that maintained the pre-pandemic (2019) classification as low-volume (<10 thyroidectomies/year), intermediate-volume (10-100 thyroidectomies/year), and high-volume (>100 thyroidectomies/year) centers during the COVID-19 pandemic years (2020-2022) in Brazil

	2019	2020	p value	2021	p value	2022	p value
Low-volume – n (variation)	258	247 (-4.3%)	0.081	246 (-0.4%)	0.822	234 (-4.9%)	0.042
Intermediate-volume – n (variation)	269	175 (-34.9%)	<0.001	178 (+1.7%)	0.628	203 (+14.0%)	0.692
High-volume – n (variation)	29	12 (-58.6%)	<0.001	15 (+25.0%)	0.555	20 (+33.3%)	0.461

Among the 258 institutions categorized as low-volume centers in 2019, 247 performed 1-9 thyroidectomies in 2020, reflecting a 4.3% reduction (p = 0.081). In 2021, 246 institutions remained in the low-volume category, representing a slight 0.4% decrease compared with 2020 (p = 0.822). In 2022, the number of institutions categorized as low-volume centers further declined to 234, reflecting a 4.9% reduction compared with 2021 (p = 0.042).

Among the 269 institutions categorized as intermediate-volume centers in 2019, 175 remained in this category in 2020, reflecting a significant 34.9% reduction (p = 0.001). In 2021, 178 institutions remained in the category of intermediate-volume centers, reflected in a modest 1.7% increase from 2020 (p = 0.628). This trend continued in 2022, when 203 institutions remained in this category, reflecting a 14.0% increase from 2021 (p = 0.692).

Lastly, of the 29 institutions classified as high-volume centers in 2019, only 12 institutions remained in this category in 2020, reflecting a substantial 58.6% decrease (p < 0.001). In 2021, 15 institutions maintained the high-volume classification, showing an increase of 25.0% compared with 2020 (p = 0.555). In 2022, the number of institutions categorized as high-volume centers increased to 20, reflecting a 33.3% increase compared with 2021 (p = 0.461) and indicating a positive trend in this category.

## DISCUSSION

Our analysis showed that approximately half of the thyroidectomies performed before the COVID-19 pandemic were carried out in intermediate-volume institutions, followed closely by low-volume centers, with only a few occurring in high-volume centers. The analysis of the surgical volume distribution showed that about 3 in 5 surgeries were performed in intermediate-volume institutions, whereas 1 in 3 and 1 in 20 were performed in high-volume and low-volume institutions, respectively. There was a notable decline in surgical volume during the pandemic, especially in intermediate-volume and high-volume institutions, while low-volume centers were less affected. In the first pandemic year, the proportions of institutions categorized as intermediate-volume and high-volume centers decreased significantly, with recovery trends observed in 2021 and 2022. In contrast, the proportions of institutions categorized as low-volume centers remained unchanged from the pre-pandemic year (2019) to the first pandemic year (2021) but showed slight reductions during the remaining pandemic years (2021 and 2022), indicating a superior resilience of these centers. Despite the smaller number of low-volume institutions, the number of thyroidectomies they performed increased, surpassing pre-pandemic levels by 2022. Conversely, thyroidectomies in intermediate-volume and high-volume institutions remained lower than pre-pandemic levels despite recovery trends from 2020 to 2022.

Previous studies established a positive link between thyroidectomy case volume and outcomes (6,8-12). Pieracci et al. demonstrated that high-volume centers, compared with low-volume and middle-volume centers, were associated with a decreased likelihood of overall complications (p = 0.005), postoperative bleeding (p = 0.010), blood transfusion (p = 0.040), respiratory failure (p = 0.040), and mortality (p = 0.004) (13).

Our study has some limitations. For example, we analyzed retrospective, aggregated ecological data from national databases. However, it is important to note that the SUS database, a widely recognized primary public data source, relies on billing information and undergoes auditing by competent authorities. Also, our analysis focused on case volumes across institutions and did not evaluate surgical outcomes or costs.

The strength of our study relies on its originality in analyzing the case volumes of institutions performing thyroidectomies in Brazil. The results provide valuable insights into the nuanced dynamics of healthcare delivery during a crisis and underscore the need for continued monitoring and adaptive strategies to maintain an effective and resilient healthcare system.

In conclusion, in summary, the COVID-19 pandemic significantly disrupted the landscape of thyroidectomies, particularly affecting surgeries in institutions with intermediate and high volumes. Conversely, low-volume institutions, which performed fewer thyroidectomies before the pandemic, displayed greater resilience during the pandemic. Although our findings are descriptive, they highlight the need for a comprehensive national database with detailed outcome information to understand further implications observed in this study and guide future research and health policy decisions regarding thyroidectomies in Brazil.
